# Secondary Education

**Published:** 1898-07-02

**Authors:** 


### The Hospital

A Journal of

The Medical Sciences and Hospital Administration.

Vol. XXIV.—No. 614.] Edited by Sir Henry Burdett, K.C.B., and Solomon C. Smith, M.D., M.R.C.P. [July 2, 1898.

### Secondary Education.

A Bill for the promotion of Secondary Education, which has been introduced into Parliament by seven members, among whom we find the name of Professor Jebb, and which is said to have been approved by the Conference of Head Masters, the Incorporated Association of Head Masters, the Head Mistresses' Association, the Conference of Catholic Schools, and the Hebdomadal Council of the University of Oxford, seems to demand the serious attention of two other not wholly unimportant sections of the community, namely, parents and ratepayers. There is, of course, no possibility that the Bill will be considered in the present session, and not much probability that it will be considered even in the next; but it deals with a subject which is likely to be brought forward again and again until some kind of legislation has been obtained, and which must be admitted on all hands to be of very great national importance. The actual proposals of the existing Bill are hardly likely to find favour with any Government. It provides for the formation of what is to be called an "Advisory Council" of forty persons, to be presided over by the Lord President or the Vice-President of the Privy Council, and to be elected by a very great number of authorities between which there is no other apparent link or community of function. The duty of this Council will be to "advise" the Education Department with regard to its methods of conducting its own business; and we doubt whether a body of permanent Civil servants will be at all inclined to submit to the sort of control which it is proposed to exercise over their proceedings. Either the Advisory Council would be permitted to cry aloud, no man regarding, or the work of the Department would be involved in folds and meshes of red tape, even more intricate and more numerous than those by which its present movements are on many occasions impeded. This, however, is only a matter of detail; and there can be little doubt of the willingness of the Minister of Education, whoever or of whatever party he may be, to extend the sway of his department over whole classes of schools which are at present independent of him.

It is alleged by the promoters of the Bill that, while much English secondary education is extremely good, much, by universal admission, is scandalously bad; and we fear this statement cannot be seriously questioned. There are many middle-class or commercial schools in which the highest aim of the proprietor is to enable his boys to pass some simple examination in general knowledge, and in which there is no endeavour either to train the judgment or to teach the pupils how to learn. Examinations, within due limits, are very good things in their way; and it is clearly desirable that they should be instituted and should be passed. It is too often forgotten, or by many has never been known, that their utility is strictly limited, and that there are numerous intellectual qualifications, of high value in the affairs of life, of which examinations furnish no criteria. The clerk or the tradesman, who sends his boy to a school suited to his station, who finds that, when there, he is well fed and comfortable, and that before leaving, to take his place at the desk or the counter, he is able to obtain a certificate showing that he has passed an examination conducted by one of the Universities, or by the College of Preceptors, is likely to be entirely satisfied. It is only those who are called upon to carry education a step farther who can realise how frequently the schooling has been worthless; and the opportunity for such realisation, perhaps, comes most frequently to the teachers of medicine or of pharmacy. The students of both are usually derived from schools somewhat more expensive, if not in other respects better, than those above referred to; and the preliminary "exam." has been the goal of the teacher's efforts. In such circumstances, exceptional ability will, nevertheless, display itself, but the ability of the average pupil or student is not exceptional, and requires to be developed along lines which examinations, and the preparations for them, do not of necessity even touch. In the famous lecture on "Education," delivered by Faraday in 1854 at the Royal Institution, and which, if it had been delivered before Haroun al Raschid, would have been "written in letters of gold, and laid up in the archives of the kingdom," the illustrious philosopher pointed out that, "in physical matters, multitudes are ready to draw conclusions who have little or no power of judgment in the cases; that the same is true in other departments of knowledge; and that, generally, mankind is willing to leave the faculties which relate to judgment almost entirely uneducated, and their decisions at the mercy of ignorance, prepossessions, the passions, or even accident." Since this was written the apotheosis of sciolism has been brought about by the cheap press; and in every railway carriage we may hear chatter about subjects which the chatterers have never tried to understand, and could not succeed in understanding if they were to try with the whole of such powers as they possess. We do, indeed, want better secondary education; and the first direction of the betterness would be in the way of teaching the limitations of knowledge, and the dangers which beset the path of ignorance.

